# Mechanical Properties and Microscopic Study of Recycled Fibre Concrete Based on Wind Turbine Blades

**DOI:** 10.3390/ma17143565

**Published:** 2024-07-18

**Authors:** Jiajing Wang, Chenghao Wang, Yongcheng Ji, Ruihang Qie, Dayang Wang, Guanxun Liu

**Affiliations:** 1 School of Aulin, Northeast Forestry University, Harbin 150040, China; zimanchengzhu@163.com (J.W.); chenghaowang1206@163.com (C.W.); qie214641@nefu.edu.cn (R.Q.); nefuguanxun.liu@nefu.edu.cn (G.L.); 2 School of Civil Engineering and Transportation, Northeast Forestry University, Harbin 150040, China; dayang@nefu.edu.cn

**Keywords:** material recovery, recycled fibre concrete, intrinsic model, recycled aggregate concrete, material microanalysis

## Abstract

In recent years, wind energy has begun to receive a significant amount of attention as clean energy is utilised and demanded in large quantities, resulting in a sharp increase in the use of wind turbines. The demand for wind turbines has gradually risen due to the clean and recyclable nature of wind energy. The current blade life of wind turbines in China is about 20 years, which means that the disposal of obsolete used blades can become a difficult problem in the future. Therefore, this study is of great significance to explore the regeneration performance of the blades after recycling and disposal. In this paper, wind turbine blades were mechanically recycled into recycled macrofibres, which were added to concrete as a reinforcing material to make wind impeller fibre concrete (WIC), and the three proportion ratios of 1%, 1.5%, and 2% were explored to compare the performance. The performance of WIC was also evaluated and its performance was compared to that of glass fibre concrete (GC). In addition, the material physical properties of second-generation recycled aggregate concrete (RAC) based on WIC were explored. The strength and peak strain variations and their causal mechanisms were analysed both macroscopically and microscopically by means of the classical mechanical tests (compression and bending tests), SEM, and XRD. The results show that the compressive strength of WIC was negatively correlated with the fibre content and increased by 6.04–18.12% compared to that of ordinary concrete (OG), with a maximum of 19.25 MPa; the flexural strength was positively correlated with the fibre content, with an increase of 5.37–18.5%. The microstructural analysis confirmed the macroscopic results and the intrinsic model better validated the experimental results.

## 1. Introduction

Wind power is a low-carbon renewable energy source. Globally, the use of wind energy has been growing and accelerating. Since 1999, wind power production in the United States has increased 30-fold, and annual installations of new wind turbines have increased 10-fold [1]. The wind power industry has grown rapidly over the last 20 years, with experts predicting that it will meet 30% of the global energy needs by the 2050s [2]. However, with the increase in power generation, many wind turbines will be retired soon, with a corresponding rapid increase in the amount of wind blade waste disposed of. By 2050, about 40 million tonnes of blade waste is expected to enter rivers [3]. One of the main challenges of this century is to embark on a path of sustainable development that balances the social and economic needs of the present with the environmental needs of future generations [4]. Currently, humanity is faced with the challenge of establishing environmentally friendly, cost-effective, and sustainable infrastructure systems [5]. In this sense, the construction industry faces a critical phase in fulfilling these three fundamental sustainability criteria. Ordinary concrete has poor strength, energy absorption, and resistance to fracture, which are its main drawbacks and areas for improvement [6]. In recent years, various fibres recycled from industrial waste have been added to concrete to minimise the concrete industry’s environmental impact. Most wind turbine blade waste comprises glass fibre reinforced polymer (GFRP), a polymeric resin, and glass fibre composite material [7]. Recycled concrete has been widely studied. Wu et al. [8] recycled waste clay brick powder as an alternative binder for cementitious materials to prepare a cement concrete with good micro–macro properties. Recycled and utilised recycled cement with recycled fine aggregate (RFA) to prepare a fully recycled mortar was studied to achieve the maximum utilisation of the concrete waste and to significantly improve the strength and impermeability properties [9]. Liu et al. [10] used ground concrete waste powder as a green binder to make a sustainable metakaolin-based polymer mortar, achieving an improved drying shrinkage of the ground polymer mortar. The recycling of materials has become a global trend, and the reuse of wind turbine blades will benefit both the economy and the environment [11]. Overall, more reprocessing options for wind turbine blades are currently available, and the recycling value does not offset the cost [12]. As a result, landfills and incineration are currently the main disposal methods for most composite wastes. Incineration has the benefit of energy recovery, but is more notable for the environmental damage caused by the release of poisonous gases during the process and the requirement of the disposal of residues [13]. Therefore, there is a need for efficient, cost-effective reprocessing solutions with a minimal environmental impact [14]. Several methods for recovering materials from wind turbine blade scrap include mechanical recovery, thermoforming, pyrolysis, and chemical methods [15]. Mechanical recycling methods for materials are efficient and economical viable. Its main principle is the continuous cutting and grinding of the material, which results in a mixture of sufficiently small pieces, such as short fibres, long fibres partially macerated with the matrix material, and clusters of material [16]. Mechanical methods differ from thermally or chemically recovered fibres in retaining surface sizing and matrix residues. This effect causes the obtained fibres to be rougher [17]. Materials derived from mechanical recovery methods are often utilised as fills, aggregates, or reinforcing materials for building [18,19,20]. For example, Yazdanbakhsh et al. [1] cut GFRP prismatic pins from decommissioned turbine blades. They added them to concrete as a partial replacement for aggregates, which significantly increased the strength. Attempts have been made to manufacture fibres by recycling them from wind turbine blade waste. Baturkin et al. [19] made a comparison of several different recycled materials from retired wind turbine blades. They concluded that recycling them into microfibres is more favourable to the performance of concrete than aggregates. Fu et al. [21] proposed the mechanical recycling of wind turbine blades to macrofibres and presented macrofibre-reinforced concrete (MFRC for short). The test conclusions indicated that the addition of fibres increased the tensile and bending strengths of MFRC by 52% and 30% compared to those of ordinary concrete, respectively. Xu et al. [22] pioneered a new mechanical method to recycle waste wind turbine blades into macroscopic fibres with a mixing length of less than 100 mm, which were used in concrete with a reduction in compressive strength by 14.07% and an increase in flexural strength by 37.85%. Yuan et al. [23] recycled glass fibre-reinforced polymer wastes by the same method to prepare MFRC, with the compressive toughness and flexural strength being significantly enhanced by the incorporation of macro-fibres.

In recent years, the share of recycled materials in cement composites has increased in the majority of advanced nations [24]. Concrete is the most commonly used material among various building materials available globally. With the rapid growth and renewal of buildings and cities around the world, concrete is consumed in large quantities. At the same time, the work on buildings or structures generates significant amounts of construction and demolition (C&D) wastes [25]. Reusing this waste in new construction will benefit the environment by reducing sand and gravel consumption and greenhouse gas emissions. After a range of comminution procedures, concrete waste can be remade into recyclable materials, which is considered an environmentally friendly and viable option for the disposal of large quantities of concrete [26]. From the perspective of construction waste, the reuse and recycling of aggregates has the practical significance of increasing availability, while also promoting sustainable development [27]. Through in-depth research and technological advances on the effects of various parameters, such as recycled aggregate quality and substitution rate on the performance of concrete, high-quality recycled aggregates that can partially or wholly replace natural aggregates in concrete have been produced [28]. Researchers have referred to the repetitive recycling of concrete as recycled concrete aggregates (RCAs), mainly natural aggregate concrete obtained by crushing concrete waste, which is used to displace natural aggregates to produce next-generation concrete, called recycled aggregate concrete (RAC). Then, this batch of concrete is crushed to generate renewable aggregates and used into the second generation of RAC [29]. In contrast to natural aggregates, the most important characteristic of RCA is the existence of residual mortar [28]. As a result, RAC is usually highly porous with weak interfacial transition zones (ITZs), which have a more significant impact on the mechanical properties [30]. Therefore, RAC has a broad development prospect and has been applied in reality. Zhang et al. [31] applied it in glass fibre-reinforced cement (GRC) wall panels with excellent performance. Moreover, there has been much research that investigates its physical performance [32,33,34,35]. Although previous research has been significant in establishing the foundations for the relevance of its use and development, RAC materials are highly stochastic and vary in their properties from source to source [27].

It has been shown that adding various types of fibres can improve the durability and different mechanical properties of concrete. Adding fibres to RAC is widely performed to enhance the compressive strength [36]. Wind turbine blade waste can be mechanically recycled to produce regenerated fibres, and although early studies noted the different forms of regenerated fibres, the main concern is still the intrinsic performance of materials made from recycled fibres. They failed to accurately characterise the mechanical properties of regenerated fibres [37]. Meanwhile, although many studies have contributed significantly to the application and development of RAC in construction, its properties vary from source to source and material, and even fewer studies have combined regenerated aggregates with wind turbine fibres, making the research in this paper more valuable and meaningful. The concrete intrinsic model, as the basis of the mechanical behaviour of concrete, connecting the theory of concrete mechanics and engineering practice, has been widely used for promoting the development of concrete engineering. The physical and mechanical properties of the internal structure of concrete are closely related to its internal structure and component distribution [6]. Therefore, compared to theoretical analysis methods, numerical simulation methods can accurately describe the internal structural properties of concrete. Based on this, it is necessary to use the intrinsic model in this paper to validate the mechanical experimental results and predict the patterns.

This study investigates the effect of recycled fibres from wind turbine blades on the enhancement in the macro–micro properties of concrete by mechanical, SEM, and XRD experiments. Compared to previous studies, the innovation of this study is to compare the strength of glass fibres recycled from wind turbine blades to that of virgin glass fibres to determine the effect of the mechanical recycling method on the properties of the fibres. This paper aims to provide a recycling method that can address the adverse environmental impacts of blade waste, provide information for exploring its optimal application, and apply it to concrete while promoting enhanced material performance and environmental friendliness in the construction industry.

## 2. Materials and Methods

### 2.1. Methodology for Recovering Fibres from Wind Turbine Blades

**Figure 1:**
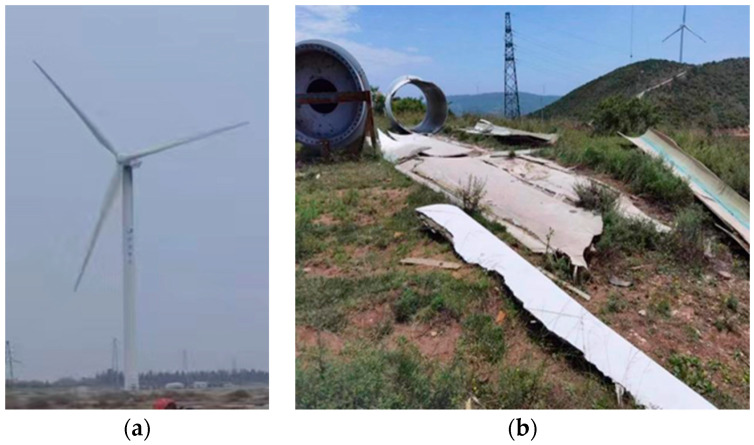
Wind power materials: (**a**) wind turbine; (**b**) decommissioned wind turbine blades.

**Figure 2:**
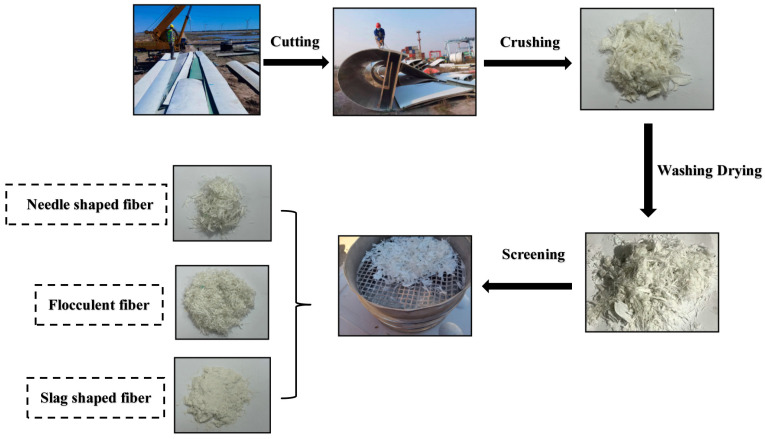
Process flow of wind blade fibre production.

The recycled fibres used in this study were obtained of retired wind turbine blades, which are shown in Figure 1. The process flow for the preparation of waste recycled fibre materials is shown in Figure 2. The blades are first cut into blocks of about one metre in the factory using diamond rope saws and then powdered, and fibrous materials are obtained by crushing and shredding. Resin and glass fibre are the main constituents of wind turbine blades, and there is a large difference in strength and the elastic modulus between them, which is revealed during the crushing process; in the same crushing conditions, the resin is quickly crushed into powder form, while the glass fibre is fibrous and the fibre length varies. Liu called the crushed material fibre-reinforced polymer (FRP), and FRP waste was screened with three kinds of screens with different apertures [37]. The FRP slag was sieved through three sieves with different apertures, and the obtained FRP slag was classified as a flake, flocculent, and powder according to its different morphological characteristics. The flocculent FRP slag is a unique form formed during crushing, with a poor dispersion and an easy-to-form agglomeration effect. On the other hand, the regenerated fibres in powdered FRP scrap are a few microns in length, making the fibrous material almost invisible to the naked eye. The production of powdered FRP slag has high requirements for crushing equipment and requires specialised crushing equipment, which makes the cost to rise significantly. Therefore, needle flake fibres were chosen as a concrete admixture in this experiment. Notably, some research has shown that wind turbine blades are almost completely recyclable, with residual powder and fine fibres that can be reused in concrete [38].

In this experiment, the FRP waste obtained after crushing was processed with stainless steel screens of 9.5 mm, 2.5 mm, and 1.25 mm size and sieved in three steps. Powdery and flocculent FRP wastes were removed, and needle-like fibres of a more uniform size were obtained. After this, about 25 percent of the total waste by weight was reused in the concrete. Manual sieving is an effective method for recovering fibres from wind turbine blade waste. However, it has the disadvantage that it is difficult to accurately determine the length, width, and thickness parameters of the mixed fibres after sieving, and it is also tricky to thoroughly sieve out substances in the waste that are detrimental to the properties of the fibres. After sieving, the obtained needle-like fibres were sent to the sink for cleaning, and fibres with different lengths, widths, and thickness parameters were produced after drying. The shorter length and lower aspect ratio of the obtained fibres are due to (1) size reduction in the parts before recycling, (2) damage of the fibres during recycling, and (3) the cutting process [39].

**Figure 3:**
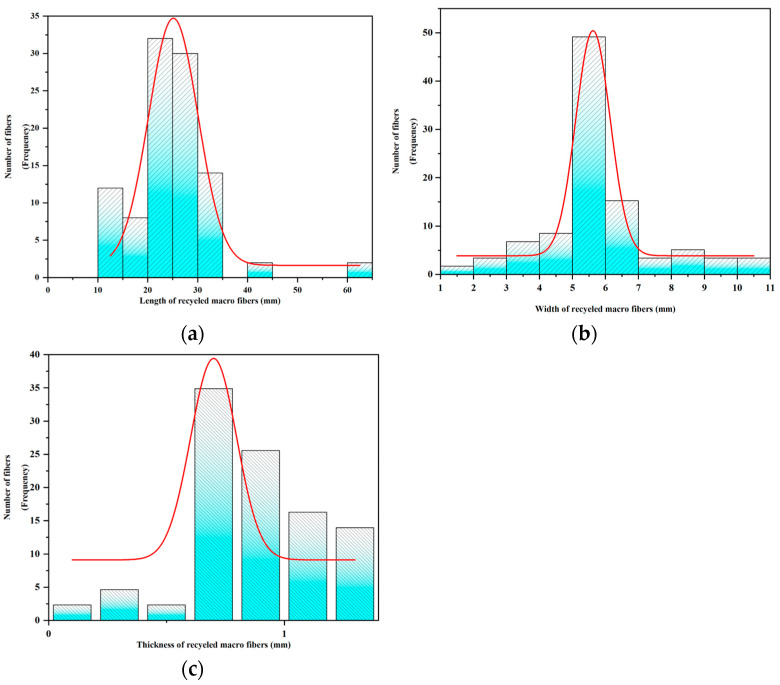
Frequency distribution of the wind blade fibre length, width, and thickness: (**a**) length; (**b**) width; and (**c**) thickness.

The largest obstacle to the efficient use of waste materials is the need for the uniformity of fibre sizes in recycling. In remanufactured composites, optimising fibre length, orientation/alignment, and the delamination of discontinuous fibres relative to stacking are critical to achieving balanced mechanical properties [40]. Therefore, in order for the physical performance of the recycled material to be improved, the larger-sized recycled materials, the screened fibres, need to be screened a second time to obtain finer fibres so that the fibre sizes of the final admixture added to the concrete are within the desired range. The selection was based on three aspects: (1) small enough to allow a sufficient amount of macrofibres to be contained in all specimens; (2) much larger than the thickness pursuing a relatively high aspect ratio; and (3) easily fabricated using machines [23]. The actual dimensions of the macroscopic fibres used in this study were measured using 200 representative samples. Figure 3 shows the fibres’ frequency distribution of the length, width, and thickness.

### 2.2. Materials and Properties

The concrete in this test consisted of fibre, cement, fine aggregates, and coarse aggregates. P·O 42.5 standard [41] silicate cement produced by Harbin Cement Plant was used for the cement; Table 1 and Table 2 list its main performance index and chemical composition. As shown in Table 3, coarse aggregates consist of 5–16 mm smaller stones and 16–26.5 mm larger stones.

The fine aggregates used in this study were NCAs (medium sand) and RCAs. Their physical characteristics obtained according to JGJ 52-2006 [42] are presented in Table 4. The recycled aggregates have a high mud content, porosity, and crushing value index due to the attachment of old mortar.

**Figure 4:**
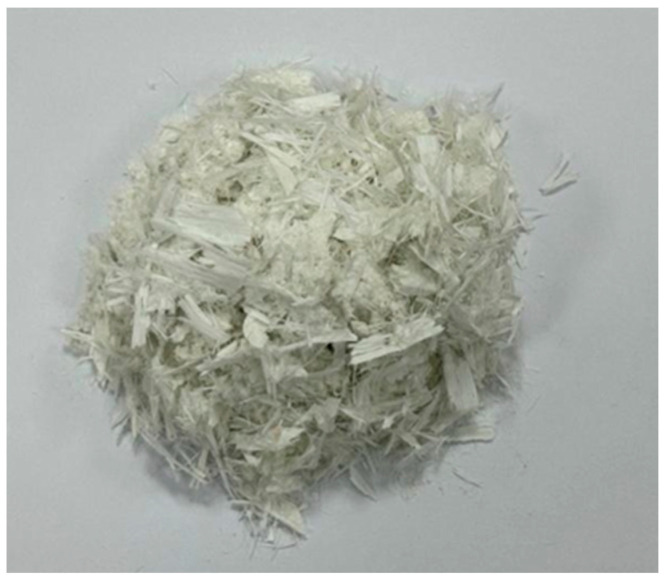
Wind blade fibres before sieving.

**Figure 5:**
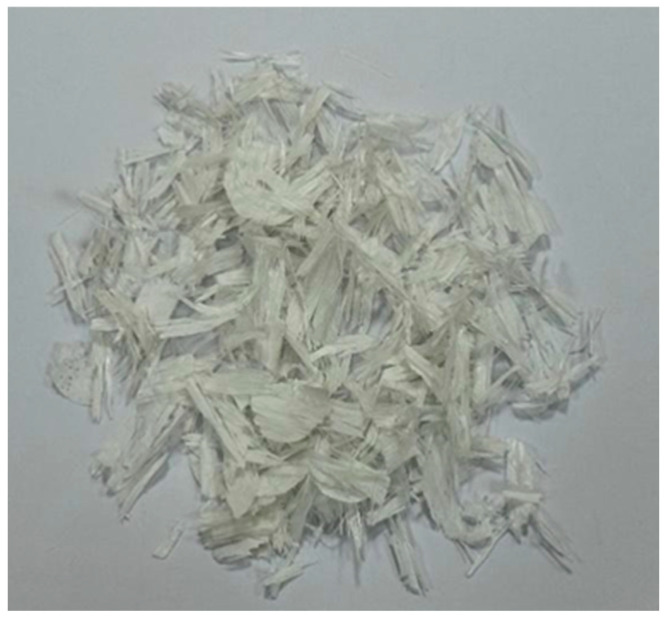
Wind blade fibres after sieving.

Figure 4 and Figure 5 show photographs of the wind blade fibres, and Table 5 shows the average statistical special. Table 6 shows some of the main physical indicators for the glass fibres utilised in the experiments.

### 2.3. Mixing Ratio Design

This study highlights the effect of recycled fibre dosage on concrete properties. Therefore, the macroscopic fibre dosage expressed in terms of the volume fraction of concrete is the critical variable, followed by the amount of recycled aggregates incorporated. In this experiment, 12 groups of concrete with different specifications were designed and tested. OG denotes the original concrete without fibres; other concrete specimens containing glass fibres or wind turbine blade fibres are denoted as GC and WIC, with the numbers indicating the percentage of the fibre volume fraction. There were six groups of OG, GC-1, GC-2, WIC-1, WIC-1.5, and WIC-2, which were classified according to the fibre types or volume fractions. At the same time, the wind turbine fibre (WTF) concretes were subdivided into six groups according to the recycled aggregate admixture (20% and 40%), with the RAC20 or RAC40 suffixes denoting the difference in the admixtures. The specific volume fractions and mix ratios are shown in Table 7 and Table 8.

### 2.4. Specimen Preparation

Prismatic concrete specimens of 400 mm length, 100 mm width, and 100 mm height and cylindrical concrete specimen moulds of 200 mm height and 100 mm diameter at the base were used for the tests. It is worth noting that, as plasticisers were not used in this test, to address the negative effect of fibre incorporation on concrete flow, fibre pre-treatment was conducted after sieve selection was completed and before making WIC: fibres were soaked in water overnight, removed, and air-dried for a short time and the surface water was wiped dry. Due to the high water absorption of the fibres, a higher water content could be obtained and WIC flowability could be maintained. The specimens were made in the following way: Firstly, all the components except fibres were mixed thoroughly; secondly, water was poured evenly into the mixture and mixed again for 2 min; thirdly, fibres were added in batches, while the mortar maintained a certain fluidity, and the mixing time was controlled to be sufficient for the components to mix. The previous pre-treatment affords the fibres sufficient moisture to enhance adhesion to the concrete, which ensures uniformity and dispersion rate; fourthly, the mixing was terminated when the mortar had a certain degree of plasticity, and it was poured into the mould for casting. After 24 h, the mould was demoulded, and it was cured for 28 d. During concrete curing, water spraying was conducted daily to maintain high humidity.

### 2.5. Testing Methods

This experiment tested the uniaxial compressive strength for cylindrical specimens and the point bending for the prismatic specimens.

**Figure 6:**
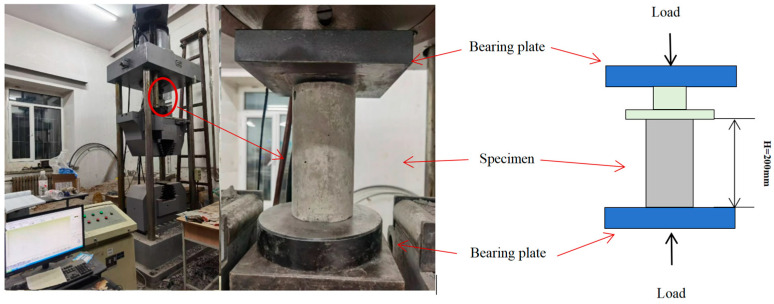
Compressive strength test.

The compressive strength of concrete was tested according to GB/T 50081-2019 [41]. The specification performs the uniaxial compressive strength test, and the loading system conforms to the standard. The full name of the machine used in this test is Microcomputer Controlled Electro-hydraulic Servo Universal Compression Tester (Shenzhen Gongyouji Group Co., Ltd., Shenzhen, China) with a static load capacity of 1000 kN, as shown in Figure 6.

**Figure 7:**
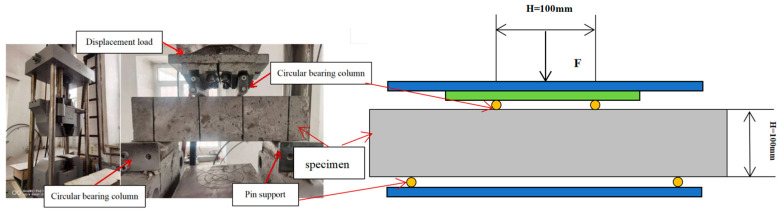
Four-point bending test.

A four-point flexural test on four short beams with the dimensions of 150 mm × 150 mm × 550 mm was used to measure the flexural strength. The test loading was carried out according to CNS1233-1984 [43], as shown in Figure 7. The concrete flexural strength averaged five 400 × 100 × 100 mm^3^ prisms with the same sample sizes and test method in each group.

This study further investigated the mechanical behaviour of WIC by analysing the stress–strain curve. The axial strain can be obtained in two ways: the ratio of the shortening of the axial end measured by the linear variable differential transformer (lvdt) to the length of the specimen (overall strain) and the average reading of the axial strain gauges at mid-height (local strain) [44]. In this test, the axial strain was determined by dividing the average of the two LVDT lengths by the scale length, and the compressive stress was equal to the applied compressive load divided by the cross-sectional area of the sample. The formula for calculating bending strength is F_b_ = P_max_ × l/(bh^2^), where F_b_ is the bending strength, P_max_ is the peak load, l is the span (300 mm), b is the width, and h is the height. When analysing the stress–strain curve of the specimen, based on the bending test results, the hysteresis energy in the loading direction was calculated as the area of each load–deformation relationship (P_D_ curve) from the origin to point (1/2) after the peak load. In this case, the deformation is the vertical displacement of the loading point. The deformation of the P_D_ curve is adjusted to the zero load point with the secant stiffness between point (1/2) and point (1/3) as the origin. The stress–strain curve of concrete can be divided into five stages: linear–elastic stage, elastoplastic stage, crack extension stage, primary crack formation beyond the peak stress point, and continuous damage stage.

In this experiment, scanning electron microscopy (SEM) was used to investigate the microstructure of WIC with different fibre contents, and the chemical composition was analysed by X-ray diffraction (XRD) detection. Blocks of concrete with a length and width of less than 10 mm and a thickness of less than 5 mm were prepared for SEM observations: the specimens after the mechanical tests were knocked off the skin and crushed with a small hammer, the flatter blocks were selected as alternative samples to be tested, and the hydration was terminated with isopropanol to prevent image artefacts, and then sent to be tested after drying. For the XRD, powdered concrete samples of 1 g each, after being ground by mortar and pestle, were used. The EMAXevolution X-Max80 was used to observe the micromorphology of the WIC specimens. The physical phase compounds were analysed by X-ray diffraction (XRD) on WIC specimens with length, width, and height lower than 5 mm under Cu-Kα radiation (40 kV, 100 mA), a 2θ angle (5~80°), and a scan rate of 6°/min. Due to the poor electrical conductivity of the concrete material, gold spraying was performed prior to the analysis.

### 2.6. Intrinsic Model

#### 2.6.1. Recycled Fibre Concrete Intrinsic Modelling

Recycled fibre concrete can be viewed as a viscoelastic material in the presence of damage, and a nonlinear viscoelastic eigen structure equation was proposed by Zhang et al. [45]. Theoretically, any published model that determines the fundamental mechanical properties of WIC can be used as an a priori model. A comprehensive database of test observations can then be applied to significantly improve model predictions [46].

(1) $$\sigma_{r}=\sigma_{e}+\sigma_{m1}+\sigma_{m2}=E_{0}\varepsilon +\alpha \varepsilon^{2}+\beta \varepsilon^{3}\;+E_{1}\int_{0}^{t}\;\varepsilon exp(-\frac{t-\tau }{\varphi_{1}})d\tau +E_{2}\int_{0}^{t}\;\varepsilon exp(-\frac{t-\tau }{\varphi_{2}})d\tau$$

where E, α, and β are the elastic constant parameters corresponding to the nonlinear elastic section of concrete. The last two integrals in Equation (2) represent two Maxwell bodies with different relaxation times, where the Maxwell body with elastic constant E_1_ is used to describe the viscoelastic response phenomenon at low-strain rates and the relaxation time is φ_1_, which is used to describe the viscoelastic response phenomenon of concrete at low-strain rates; Maxwell bodies with an elasticity constant of E_2_ are used to describe viscoelastic response phenomena at high-strain rates, using the relaxation time of φ_2_. Damage is a factor that must be considered when studying the physical–mechanical characteristics of concrete materials, and the coefficient of damage (or damage degree) D is a very prominent internal variable in the concrete constitutive model materials. Given that the effect of damage on the physical and mechanical characterisation of concrete materials is all-encompassing, the dynamic ontological relationship of concrete materials should include the internal variable of damage factor D. Therefore, the constitutive equation should be written as follows.

(2) $$\begin{array}{ll} & \sigma_{a}=\sigma_{r}(1-D)=(1-D)(\sigma_{e}+\sigma_{m_{1}}+\sigma_{m2})\\ = & \left(1-D)(E_{0}\varepsilon +E_{1}\int_{0}^{t}\;\varepsilon exp(-\frac{t-\tau }{\varphi_{1}})d\tau +E_{2}\int_{0}^{t}\;\varepsilon exp(-\frac{t-\tau }{\varphi_{2}})d\tau \right) \end{array}$$

where σ_a_ is the apparent stress and σ_r_ is the stress when the material is undamaged.

#### 2.6.2. Damage Factor Equation Fitting

In order to study in depth the impact of wind blade fibres and the recycled aggregate substitution rate on the mechanical characteristics of concrete under uniaxial compressive loading, the destruction process of fibre-reinforced concrete is regarded as a continuous development process, and the following assumptions are made: (1) The recycled coarse aggregates and wind blade fibres are uniformly distributed in concrete; (2) The content of crushed concrete blocks in recycled coarse aggregates in different concrete specimens is not necessarily the same (the reason for the composition of recycled coarse aggregates not being uniformly distributed); (3) When the recycled concrete is damaged under pressure, the ITZ between the recycled aggregates and cement paste firstly appears damaged, and the ITZ between the natural aggregates and cement paste also appears damaged in the process of damage development, and the two kinds of damage develop at the same time, which leads to the destruction of the concrete; (4) The ITZ between the recycled concrete and the wind blade fibres is regarded as consisting of countless interfacial microelements, and the interface has preliminary damage in the process of damage development. Regarding the composition of the interface, there is initial damage before the linear–elastic relationship, while the interface microelement strength obeys the Weibull distribution. At the same time, due to the wind power fibres being similar to glass fibres, which have an apparent fibre enhancement effect, the stress–strain curve shows an obvious damage softening behaviour, which is the macroscopic response to the generation of micro-cracks within the material; the expansion of micro-cracks occurs until the convergence of the microscopic process, and the Weibull distribution is particularly suitable for describing the fracture damage process of materials. Dong [47], Ji [48], and Zhang [45] used the Weibull distribution of the amount of damage in the intrinsic study of concrete and fibre-reinforced composites to describe the nonlinear behaviour of the experimental stress–strain curves. They achieved results that were very much in line with the expectations.

According to Lemaitre’s principle of strain equivalence [49], the value of the strain of the effective stress acting on the undamaged material is equal to that of the strain of the nominal stress acting on the damaged material, which the following equation expresses:

(3) $$\varepsilon =\frac{\sigma^{*}}{E}=\frac{(1-D)\sigma }{E}$$

where ε is the strain; σ* is the nominal stress; σ is the effective stress; and D is the damage variable.

The ratio of the number of microelements at the damaged interface to the total number of microelements at the interface is defined as the damage variable, and the development process of the concrete damage variable is separated into two phases.

The first stage of the calibration of the damage variable is the initial damage, which is conducted by the mixing in the recycled coarse aggregates and improves with the increase in the amount of recycled coarse aggregates. From the theory of damage mechanics [50], it is known that the macroscopic mechanical property response of the concrete material characterises the degree of deterioration within the material; so, the preliminary damage variable D_R_ of fibre-recycled concrete with substitution rate R is:

(4) $$D_{R}=1-\frac{E_{R}}{E_{0}}$$

where E_0_ is the modulus of elasticity of concrete, with a 0% replacement rate; E_R_ is the modulus of elasticity of concrete and varies with the substitution rate R; and D_R_ is the preliminary damage variable of the concrete due to fibre incorporation.

Stage 2 damage variables are mainly developed during the loading stage of fibre-recycled concrete. From the principle of strain equivalence, the constitutive relationship of recycled concrete with the replacement rate R is:

(5) $$\sigma =E_{R}\varepsilon (1-D_{C})$$

where D_C_ is the load-induced damage variable, which is expressed by Equations (4) and (5):

(6) $$\sigma =E_{0}\varepsilon (1-D_{C})(1-D_{R})$$

(7) $$D=D_{R}+D_{C}-D_{R}D_{C}$$

(8) $$D_{C}=\frac{n}{N}$$

where D is the total damage variable; and the load damage variable D_C_ is the proportion of the quantities of interface microelements damaged under load n to the total quantity of interface microelements N. The probability density function of the interface microelement strength is as follows.

Since the strength of interface microelements obeys the Weibull distribution, Equation (9) is in accord with its probability density function.

(9) $$\varphi (F)=\frac{b}{a}{\left(\frac{F}{a}\right)}^{b-1}\cdot exp[-{\left(\frac{F}{a}\right)}^{b}]$$

where F is the distributional variable of the strength of the interface microelement; a and b are numerical parameters of the Weibull distribution; and the relationship between the proportion of damaged interface microelements, n, and the total of interface microelements, N, under a certain strain condition is expressed as follows:

(10) $$n=N\int_{0}^{\varepsilon }\varphi (\varepsilon )dF=N\{1-exp[-{\left(\frac{\varepsilon }{a}\right)}^{b}]\}$$

Substituting Equations (4) and (7) into Equation (9), the damage ontological model of recycled concrete at various replacement rates can be attained:

(11) $$\sigma =E_{R}\varepsilon exp[1-{\left(\frac{\varepsilon }{a}\right)}^{b}]$$

Combining Equations (3) and (5), the total damage variable D can be obtained for recycled concrete with different substitution rates:

(12) $$D=1-\frac{E_{R}}{E_{0}}exp[-{\left(\frac{\varepsilon }{a}\right)}^{b}]$$

To explore the effectiveness of the Z-W-T principal model and the Weibull damage model in fitting the stress response, the results of the stress–strain curve tests and simulations were verified in this research, and the results are shown in figure in the last part.

## 3. Results and Discussion

### 3.1. Compressive Strength

**Figure 8:**
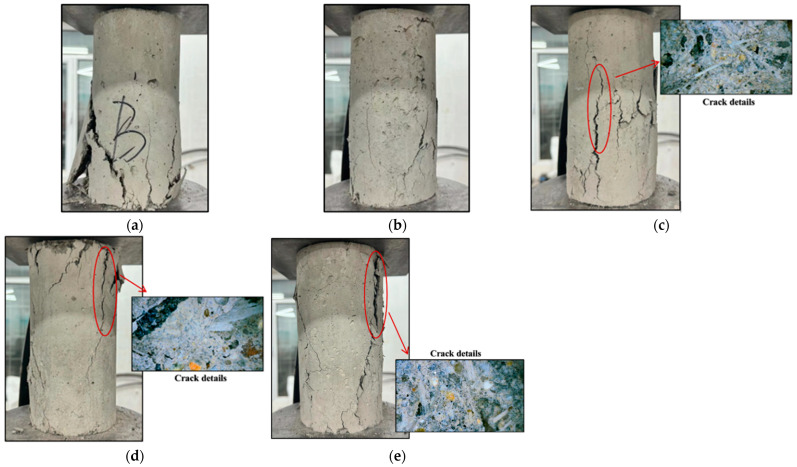
Compressive damage pattern and fibre microdistribution of cylindrical specimens: (**a**) OG; (**b**) GC-2; (**c**) WIC-1; (**d**) WIC-1.5; and (**e**) WIC-2.

Figure 8 depicts the typical damage pattern of the cylindrical specimen under compression conditions. The control specimen (OG) was severely crushed. It showed a typical conical damage pattern with several large spalls (Figure 8a). In the fibre concrete specimens, the inclusion of fibres inhibited the cracking of the concrete. As a result, the glass fibre concrete specimens retained their integrity better after damage. They did not break into fragments (Figure 8b). The concrete specimens with wind blade fibres also spalled significantly less than the plain concrete specimens. The damage of specimens with low volume ratios of wind blade fibre (e.g., 1% admixture) showed a typical shear-type fracture damage pattern (Figure 8c). In contrast, the loading damage on the high fibre admixture concrete (WIC-2) produced vertical cracks along the loading direction, and due to the pulling effect of the internal fibres, the concrete spalled less severely and had a better integrity compared to the low admixture specimens (Figure 8e). However, the top region of the specimen WIC-1.5 spalled as shown in Figure 8d. The microscopic phenomenon of the compressive fracture surface in Figure 8 shows that the number of fibres in specimen WIC-1 was low, but the distribution in the concrete was relatively uniform. In addition to this, some fibres were detached from the cracks. These fibres connected the two fracture surfaces and intersected with them simultaneously when the cracks were expanding. Thus, these bridging fibres mitigated the further development of the cracks. The fibres were unevenly distributed in the specimen of WIC-1.5, which follows the objective law that macro-fibres are more challenging to distribute uniformly in cylindrical specimens than microfibers. Thus, it does not provide sufficient reinforcement for all areas [51]. This explains this specimen’s severe damage and possibly the compressive strength law summarised later in the article. The WIC-2 specimen section also showed an uneven distribution of fibres, despite the high number of fibres and bridging effect. Also, the mechanical recycling process of the wind turbine fibres resulted in a characteristic rough surface of the fibres mixed with magazines, and manual sieving did not eliminate the components that were harmful to the compressive strength, which may have reduced the compressive strength of the high admixture specimens compared to the low admixture specimens. Overall, wind blade fibres in the concrete act as bridges in cracks, restraining the relative sliding of the macroscopic shear crack surfaces, thus impeding crack extension and a more ductile damage process [21]. This trend is more pronounced as the fibre content increases.

**Figure 9:**
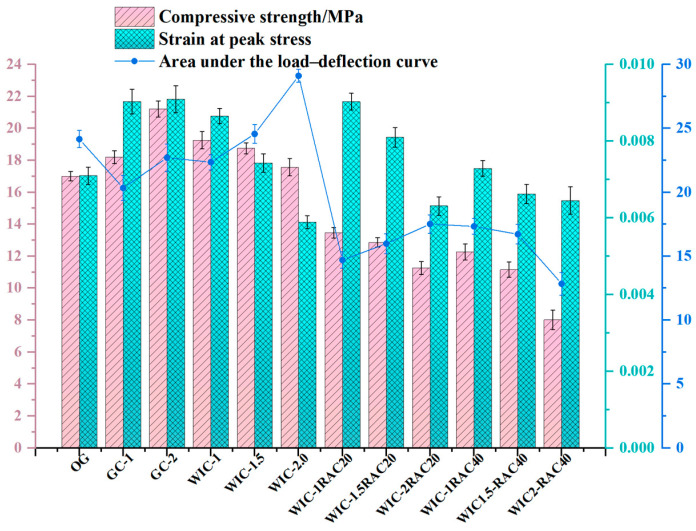
Compressive test results.

Table 9 shows the main results of the compression tests. The dosage of recycled fibre was 0, 1 wt%, 1.5 wt%, and 2 wt%; from the graph, it can be observed that, as the dosage of recycled fibre increases, the compressive strength of WTF concrete decreases gradually, but the lowest value is still higher than the highest value of plain concrete. The greatest increase in compressive strength is achieved when 1 wt% of recycled fibre is added, up to 19.25 MPa. With the increase in the admixture of glass fibre, the compressive strength is improved, and when the admixture is 2 wt%, the compressive strength is increased up to 17.56 MPa. As shown in Figure 9, comparing the wind blade recycled fibres laterally with the highest compressive strength values of glass fibre concrete (2% glass fibres and 1% wind blade fibres), the glass fibre concrete performs better. The shorter and more homogeneous glass fibres have a higher degree of dispersion in the concrete, which enhances the transfer of load to the fibres and thus increases the compressive strength of the specimens. It is essential to note that the recycled fibre edges were not subjected to any chemical treatment that would otherwise increase the cost of production of the material, and therefore, maybe more rough and uneven and accompanied by impurities compared to glass fibres, which could to explain the observation of this law [52]. From the longitudinal point of view, the compressive strengths of 1.0%, 1.5%, and 2.0% WTFE concrete were 19.25 MPa, 18.75 MPa, and 17.56 MPa, respectively, which are an increase of 18.12%, 13.22%, and 6.04%, respectively, compared to the control sample, with the highest strengths being obtained at the lowest admixture levels. This observation is comparable to the phenomenon investigated by Yazdanbakhsh et al. [1], where the compressive strength increased at a lower dosage of FRP needle treatment compared to the control specimens treated without FRP needles. However, the further increase in the use of fibre glass needles led to a decrease in the lifting range. This is similar to the pattern of Xu et al. [22]: when the fibre volume ratio is higher (2.5%), the reduction in the compressive strength of concrete with recycled macro-fibres is 14.07% lower than that of normal concrete. The reasons for the different compressive strengths of concrete with different fibre admixtures are analysed as follows:

1. Low admixture: Macrofibres in concrete have three roles: (1) absorption of little free water, leading to a slight reduction in the water–cement ratio; (2) to restrain the development of cracks; and (3) to introduce a weak interface between the macrofibres and the concrete [21]. In general, if the compressive strength is increased, it should be attributed to the first two effects being stronger than the third; otherwise the strength decreases. When the wind blade fibres are kept at a low admixture (1%) during the fracture process, the needle-like wind blade fibres break due to compressive stresses, and the fibres are distributed longitudinally and horizontally in the concrete, which plays the central reinforcing role. However, the reinforcing effect of wind blade fibres on concrete is also related to interfacial bonding, and the macroscopic properties are analysed from the direct interfacial bonding of the components in the composite. The wind blade fibres are firmly embedded in the cement matrix, and the strongly bonded interface can transfer the stresses to the high-tenacity glass fibres when subjected to external forces, thus increasing the overall strength of the WIC [38].
2. Medium dosage: The length of the WIC fibres selected in this experiment was within a specific interval, which was graded to play the role of skeleton support and filler, a better qualitative choice. At the same time, the dosage was not higher, but better; when higher than the appropriate range, too many fibres agglomerate and gather together dramatically impacting the compressive strength [53].
3. High dosage: When the dosage is too high, the fibres inside the stress surface are inevitably arranged longitudinally and transversely, and when there are almost no fibres inside the stress surface, the compressive strength is reduced due to internal defects resulting in a reduction in the compressive strength. With the increase in stress, when the direction of the fibres in the matrix is the same as the direction of crack generation, it leads to the further extension and expansion of cracks, which coupled with the doping of fibres produces more pores. The higher the porosity, the faster the decrease in the performance of the composite material, resulting in a rapid decrease in the compressive strength, and the compression curve of the machine test decreases rapidly. Thus, the specimen can be easily crushed [53].

Although the compressive strength pattern of the fibre test group can be derived from the data, the test results of individual specimens are different compared to the same group. These phenomena may be due to the fact that the concrete force area is 100 mm × 100 mm, and the indenter generates more pressure on the force surface in the landing process. Regarding the admixture process, the fibres do not lay flat in the matrix as in the grid structure during the admixture process but show disorder within. Due to the uneven dispersion of the waste recycled fibres within the matrix, even the doping amount may lead to different properties. However, this phenomenon exists by chance and does not affect the general pattern.

Figure 9 shows the test results for the RAC test group. The compressive strength decreased significantly with the increase in the recycled aggregate substitution ratio. At the same fibre substitution ratio, NAC had a 2–10% higher compressive strength than RAC. These results are consistent with the findings of many other researchers [26]. The reasons can be attributed to the following two points. Firstly, RCAs are less active than NCAs, and few hydration products are formed in the cement matrix; so, there is no strengthening effect. Compared to NCAs, RCAs have the disadvantages of lower strength, lower density, more surface porosity, higher water absorption, and weaker bonding ability to new mortar. The difference in the modulus of elasticity of the aggregate and the cement paste leads to tensile stresses. The internal stresses increase with the increase in the aggregate particle size, and this can adversely affect the strength of concrete [54]. This supports the results of many studies that showed that the application of RCAs may lead to a decrease in strength. Secondly, the size of the RCAs is likewise an important factor in the loss of strength. Usually, using recycled aggregates with a smaller particle size enhances the continuity of the internal structure of concrete and improves the bonding between the aggregates and cement paste. On the contrary, using RCAs with a larger particle size increases the porosity, which leads to a loss of strength. The recycled aggregates selected in this experiment had a large particle size, an essential reason for the decrease in strength [26]. The results show that the compressive strength of RAC is significantly affected by the RCA admixture to some extent. It is worth noting that, in the study by Kim [26], recycled aggregates were recycled to investigate their effect on mechanical properties. The higher the number of cycles, the more obvious was the loss of mechanical strength. This suggests that RCAs derived from repeated RAC are detrimental to the strength of concrete, which is becoming increasingly significant. This provides testimony to the conclusions of our test.

**Figure 10:**
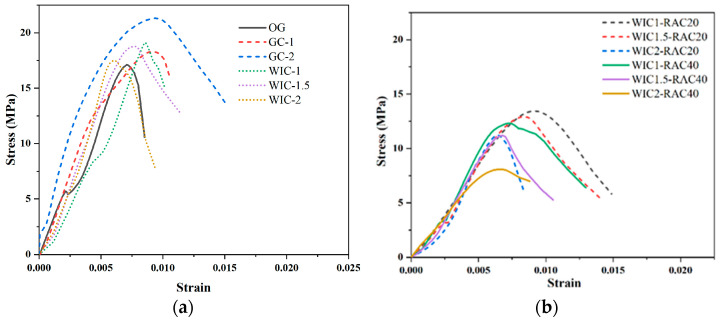
Compressive stress–strain curve: (**a**) natural aggregate fibre concrete; (**b**) recycled aggregate fibre concrete.

Figure 10a shows the compressive stress–strain curve of the specimens. As it can be seen in the figure, even though there are various types of differences in the variables, the differences in the rising section of the curves for all the specimens are minimal, indicating that the effect on the modulus of elasticity of concrete is negligible when fewer fibres are incorporated (≤1.5%). The initial slopes of the curves for the WT fibre specimens were slightly smaller than those of the corresponding control specimens. Figure 10a shows that the peak stress of the control was 16.15 MPa, and that of the wind blade fibre specimens (1%, 1.5%, and 2%) were 19.27 MPa, 18.95 MPa, and 17.87 MPa, respectively, resulting in an increase in peak stress by 10.5–19%. Similarly, the peak stresses of glass fibre specimens were 18.26 MPa and 21.25 MPa, 13–31.57% higher than those of the control. The peak axial strain of the wind blade fibre specimen was 0.0086, and that of the glass fibre specimen was 0.0094, a 22.85% and 34.3% improvement, respectively. Therefore, incorporating the appropriate content of needled wind blade fibres into concrete can enhance its compressive strength. However, the peak of performance enhancement is slightly weaker than that of glass fibre as the wind blade fibre is recycled. The presence of wind turbine fibres can delay the onset of cracking. As a result, a considerable enhancement in toughness is observed, similar to the pattern described in the literature [55]. In addition, the specimens containing wind blade fibres exhibit a comparable compressive ductility to those containing glass fibres at the same fibre content. This is because the fibres bridge the airfoil cracks, thus creating a smoother downward segment. Generally, an increased fibre content leads to a more gradual softening process in concrete [21]. A similar phenomenon was observed in the BFRP pin test by Dong [44], where the BFRP pin group specimens showed a more extended smoothing period after the curve decreased from the peak point compared to the control ssgc (seawater sea-sand gravel concrete) specimens. In addition, as it can be seen in Figure 10a, the ultimate stress and ultimate strain of the wind blade fibre specimens were significantly increased compared to those of the control specimens, and the maximum increase was observed at a doping level of 1%. The compressive deformation and tensile action of the needle-like wind blade fibres also explain this result.

Figure 10b shows the stress–strain curves of RAC. It is clearly observed that the slope of the rising section is larger for the NAC compared to the RAC. When the WIC fibre content is constant, there is a significant difference in the curve characteristics of the RAC with different RCA substitution ratios. The slope of the ascending branch varies significantly with the increase in the substitution ratio. The compressive strength of the NAC is higher than that of the RAC. At the same fibre dosage, the fc decreased with the increase in the RCA content, mainly attributed to the porous bonding mortar on the RCAs. In the case of the same RCA content, the fc tends to increase and decrease with the increase in the fibre dosage. For the 1% WIC fibre admixture, the curve of the rising branch of the recycled aggregate concrete surrounds the curve at other dosages with the highest peak stress. Comparing the downward segments of the other curves, the descending branches at the 2% and 1.5% WIC admixtures are steeper and relatively more brittle than the descending branches at other dosages. The phenomenon that the strength of RAC can be reduced is attributed to two types of ITZs in the matrix. In NAC, ITZ occurs between mortar and aggregate. In contrast, the ITZ in RAC occurs between the old and new mortar and the original aggregate. In addition, to achieve the desired workability, in many cases, a large amount of water is used in RCA concrete mixtures, contributing to the lower compressive strength. Old mortar on the surface of the RCAs reduces the compressive strength of the RAC because it has a lower density [56,57,58].

### 3.2. Flexural Strength

**Figure 11:**
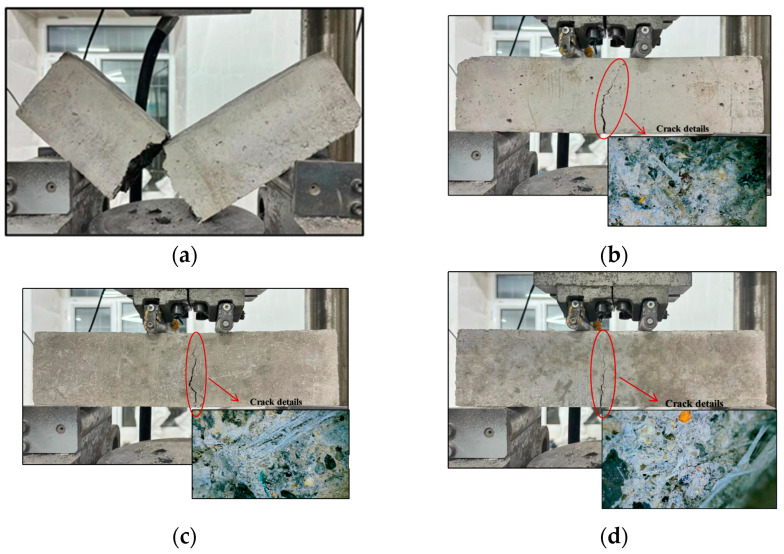
Flexural damage patterns: (**a**) OG; (**b**) WIC-1; (**c**) WIC-1.5; and (**d**) WIC-2.

Figure 11 shows the damage patterns of the prismatic specimens after four-point bending tests. All samples failed due to the formation and development of the main flexural crack in the constant moment area [21]. OG abruptly fractured into halves at the location of the main crack and exhibited brittle damage (Figure 11a). On the contrary, the specimen with fibres withstood the post-cracking bending load to remain intact without breaking in half. As the mid-span deflection increased, the crack continued to progress upwards. Due to the bridging action of the internally randomly distributed fibres in the cracked section, severe cracking occurred but did not break in half. Note that, for the 1% wind blade fibre specimen, the main crack continued to expand upwards after cracking and eventually reached the top surface of the specimen (Figure 11b). According to the literature [23], this is due to the relatively low-fibre content and limited bridging action, which approximates brittle damage. The damage mode of the specimens reinforced with WT and glass fibres was ductile. The load continued to increase after the appearance of the main crack and decreased after the peak. The sound of fibres breaking or pulling out could be heard during the fracture process, and some fibres still connected the two halves of the specimen after the damage. The damaged specimens were fractured along the cracked surface to check the distribution of fibres. In several groups of specimens in this test, generally speaking, the specimens with a good crack resistance had a more uniform distribution of fibres within the specimen. The WIC-1 specimen had fewer fibres, was severely damaged, and had a weaker strength enhancement effect. However, the needle-like WIC fibres were firmly embedded in the cement matrix, and the strongly bonded interface can transfer the stresses to the high-tenacity fibres when concrete faces external forces, thus increasing the overall strength. The WIC-1.5 specimen in the figure had a much larger number of fibres, which were more evenly distributed; thus, a further increase in strength may have been achieved. The observation of the prismatic WIC-2 specimen showed a uniform distribution of WIC fibres on the fracture surface, forming a three-dimensional architecture with a tight bond to the matrix.

**Figure 12:**
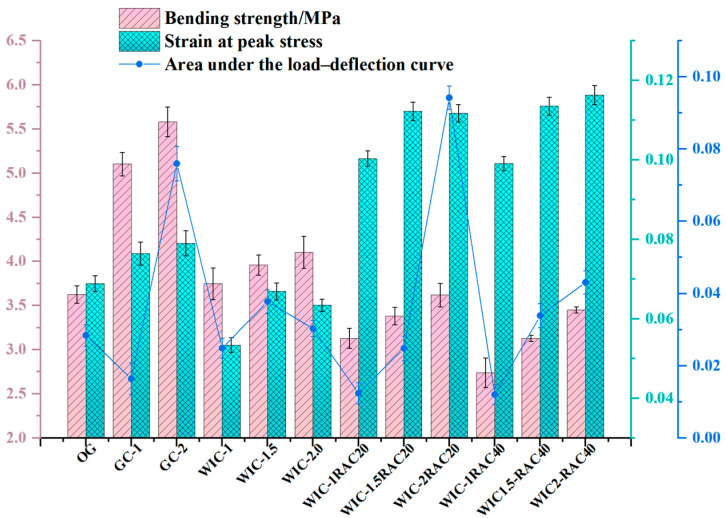
Flexural test results.

The main results of the four-point flexural test are shown in Figure 12. It can be seen that the flexural strength of the concrete increased with the addition of GC-1, GC-2, WIC-1, WIC-1.5, and WIC-2, while the flexural strength with the addition of GC-2 was 1.43 MPa higher than that of WT2. The addition of recycled fibres was evident in this range of the reinforcement of the recycled fibres in the matrix. The flexural strength increased with the amount of the admixture, and at the admixture level of 2 wt%, the flexural strength reached the highest value. The results show that the flexural strength of WIC is significantly improved. The addition of wind blade fibres improves the flexural properties, suggesting that recycled fibres recovered from decommissioned wind blades can be used as discrete reinforcement for concrete. It should be noted that the incorporation of wind blade fibres significantly improved the scatter of the test data, as the more significant the volume of individual fibres, the smaller the number of fibres, which undoubtedly increased the randomness of the fibre distribution.

Wind blade fibres are made from glass fibres cured with resin. The wind turbine blade waste contains many glass fibres, and after milling, some of these glass fibres can be stripped from the resin and broken into finer fibres, enhancing the strength of the matrix. In Figure 12, when the amount of wind blade fibre substitution is small, only 1%, the flexural strength of the specimen is slightly enhanced by 5.37%, and when the amount of wind blade fibre substitution is increased to 2%, the flexural strength is the largest, which is 4.15 MPa and is 18.5% higher than that of the control specimen. This fits with the results obtained by Xu et al. [22], where the flexural strength increases when the fibre dosage is increased, even though the compressive strength decreases. This can be explained by the fact that, when the wind blade fibre doping is too low, the glass fibre content in it is low, which only slightly counteracts the adverse effect of the FRP waste on the flexural strength properties; when the substitution amount is increased, the glass fibre content in the blade waste increases and plays an excellent toughening role [59]. The fibres have high toughness and strength and can form a interlocking network structure. The load applied can be dispersed and transferred through the fibres inside the specimen, and the corresponding flexural capacity increases. In addition, the recycled fibres have a resin coating on the surface, which contains a large amount of CaO, Al_2_O_3_, and SiO_2_, which are the nucleation points for the formation of C-S-H gels that can provide additional slip resistance at the fibre–cement interface, limiting the development of cracks and improving the interlocking properties of the fibres and cement [60]. The incorporation of glass fibres can significantly improve the flexural properties of concrete, which has been well demonstrated in previous studies. Glass fibres at 1% and 2% increased the flexural strength by 40.6% and 51.7%, respectively. With the same 2% admixture, we found that the flexural strength of glass fibre concrete was higher than that of wind blade fibre concrete. The reason for this may be explained as follows: When the fibre amount of WIC is too high, it tends to agglomerate during mixing with water and forms defective areas in the concrete, thus reducing the flexural properties. At the same time, the number of glass fibres is higher, and the fibres have split filament brooming, which can wrap the matrix like a fishing net and overlap each other to connect the matrix material so that the concrete can effectively distribute the loads received and improve the mechanical properties [53].

The high admixture of wind blade fibres can increase the flexural strength of concrete substantially; this may be because, when concrete faces external force, the fibres can absorb part of the external energy so that the matrix of the micro-cracks expands slowly. When subjected to strong external forces, the matrix fractures, but the fibre is undamaged, has no fracture, is still closely connected to the matrix, and is not pulled out of the matrix; when the matrix is broken into two pieces, the fibre is pulled out of the cement adhered to the top of the fibre and fibres regenerate to enhance the concrete impact resistance, thus improving the mechanical properties, increasing the flexural strength, and reducing the brittleness of concrete. When cracks are generated, the cracks and the recycled fibres cross each other. The fibres play a role in transferring the loads so that the stresses at the intermediate end of the cracks are dispersed. The extension of the crack length is blocked, thus weakening or eliminating the external stresses [53]. This leads the higher wind blade fibre admixture to further increase the flexural strength of concrete. The tight interfacial bond between the wind blade fibres and the concrete disperses most of the stresses and produces the desired reinforcement, which proves the high feasibility of using WIC as a reinforcing material.

The results of the flexural strength of RAC are shown in Figure 12 and Table 10. The flexural strength of RAC follows a similar trend as the compressive strength, decreasing with the increase in the RCA content. The peak flexural strength of the test group occurs at 3.62 MPa for WIC-2% and the 20% recycled aggregate dosage, which is 20.59% lower at the same WIC fibre dosage than that of NAC. Overall, increasing the RCA content from 20% to 40% reduces the increase in strength from 13.89% to 20.59%. This deterioration is due to many ITZs in RCAs with a lower tensile strength and some micro-cracks in the old mortar in the RCAs during fracture, resulting in a lower flexural strength [25].

**Figure 13:**
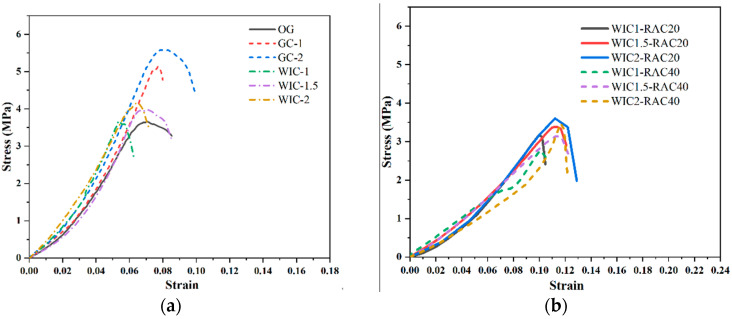
Flexural stress–strain curve: (**a**) natural aggregate fibre concrete; (**b**) recycled aggregate fibre concrete.

Figure 13a shows the stress–strain of the prismatic specimens. The pre-cracking state of all the samples is presented as a linearly increasing segment in the curve, and they all have almost the same stiffness. However, after the appearance of the main crack, the fibres start to be motivated to resist the force of the stress and act as a bridge to the crack, causing a further increase in stress followed by a large deformation after the peak stress. Similar to the pattern described in the literature [61], all the specimens showed significant descending branches with a good fracture toughness after reaching the peak point as the fibre volume substitution ratio increased. However, there were significant differences in the downward segments of all the samples, and the main reason should be the random character of the cracking behaviour of the concrete as well as the number and distribution of the fibres in the main cracks. According to the literature [21,55], the curves are mainly classified into three typical types: Type I is the control type with only one ascending section. It is characterised by a sudden drop in stress to 0, once the main bending crack appears. Samples with this feature are characterised by a sudden drop in stress, which results in the unavailability of the post-peak curve. Type II, such as wind turbine fibre 1.5 and glass fibre 1, has a rising section at the front, which is followed by a fast strain-softening section-Type III, in comparison to other groups containing wind blade fibres and glass fibres, uniquely shows an ascending section, then a strain-hardening section, and then a strain-softening section. The increase in the fibre content from 1% and 1.5% to 2% for glass fibre concrete and wind blade fibre concrete resulted in a change in the curve type from Type II to Type III, which indicates a significant increase in the flexural strength and ductility of the concrete. The stress–strain curve is of Type II for 1% and 1.5% WIC fibre concrete. When the main bending crack is created, some of the tensile force exerted on the lower concrete is released. Due to the fibre bridging action, the fibres on the cracks absorb this part of the tensile force. However, if the fibre content is low, the number of fibres will be insufficient to support this process. Therefore, these fibres are either gradually dislodged from the concrete or ruptured immediately after the appearance of the main crack. The stress–strain curves for glass fibre concrete and specimens with a high wind blade fibre content (i.e., 2% wind blade fibres) were of Type III, showing a linear branch that hardens and then rises. In these specimens, the relatively large number of macroscopic fibres passing through the cracks indicates that they resisted the full tensile force released by the cracked concrete and could allow further increases in the applied stress. After the peak stress, the fibres that passed through the cracks began to shed or break, forming the softening branch of the stress–strain curve. Figure 13a shows that the WTF concrete specimen curves are more significant than the scatter of the glass fibre concrete specimen. This can be interpreted to mean that there are more drawbacks in adding more wind blade fibres than glass fibres and, in addition, it may not be possible to attain a near-perfect random distribution of the fibres. The effect on the curves was more pronounced when varying the fibre dosage (1%, 1.5%, and 2%) in the wind blade fibre test group compared to the plain concrete, and the peak stresses and strains of the curves were significantly higher as the dosage was increased. Compared to OG, the peak stresses of WIC were increased by 2.9%, 8.5%, and 14.1%. The low-dosage specimens showed only a small increase, which the insufficient bridging effect of the fibres can explain. On the contrary, enhanced flexural properties are observed in the curves after increasing the dosage due to adequate fibre-bridging. The above discussion shows that the addition of wind turbine blade fibres can improve the flexural properties of concrete.

Figure 13b shows the whole cyclic compression process of recycled aggregate concrete specimens experienced five stages: no obvious crack extension (the bridging effect of the wind blade fibres was not obvious); obvious longitudinal fine cracks appeared on the surface of the specimen; many fine cracks appeared on the surface of the specimen and developed rapidly; when the curve exceeded the peak stress point, prominent diagonal cracks began to form as the fine cracks crossed collectively and the fibres gradually pulled out of the concrete matrix; and the continuous damage stage. As the number of load cycles increases, the fibres are pulled out of the concrete matrix and are able to act as bridging fibres in quantities insufficient to support the load capacity of the specimen. Due to the anchoring effect of the fibres, the matrix is not dispersed, although the concrete is fragmented. The damaged surface, consisting of pressure and shear, expands, eventually forming a continuous crack with a decreasing stress–strain curve. Overall, the stress–strain curve increases proportionally at the beginning of loading, and the curve is approximately a straight line. There is plastic deformation of the concrete specimens as the stress increases, and the slope of the curve slows down. After reaching the peak load, the curve of RAC decreases fast, and more cycles lead to a gradual convergence of the residual stress to 0 MPa, which is an obvious phenomenon of brittleness. It can be seen that the compressive strength of the RAC specimens decreases as the replacement rate of recycled aggregates increases. After reaching the peak point, the stress of ordinary RAC decreases rapidly, but the specimen always has a certain load-bearing capacity due to the addition of wind turbine blade fibres. We observed the law that an increase in the fibre content slows down the decrease in the load-bearing capacity, and the specimens show stronger ductility characteristics. This is due to the fact that the load-bearing capacity of the specimen after the peak point is composed of frictional resistance and residual bonding between the matrix and fibres [36].

### 3.3. Microscopic Characterisation

#### 3.3.1. SEM

After several sieving operations when making the samples, the fibres were in a separated state of refinement. The main components of the wind turbine blades include glass fibres and epoxy resin [62]; so, some small particles of non-uniform shape and size were present on the surface of the recovered fibres. Tiny signs of broken polymer resin remained adsorbed on the surface, and the acicular fibres in the macroscopic state presented resin-coated fibre bundles in the microscopic state. According to the literature, epoxy resins have the main characteristic of having multiple epoxy groups per molecule, groups that react very readily with proton donor substances, such as PLA [63]. Therefore, good interactions can be formed between the functional groups of PLA and epoxy resins through hydrogen bonding. This reaction allows for the extension of the molecular chain, thus improving the interfacial strength between PLA and the wind blade fibres and enhancing the mechanical interaction. The figure shows that the fibres are present with a small amount of resin attached to the surface, mainly appearing as an irregular powder. Notches and narrow lanes of different widths and sizes are distributed throughout the fibre axes, and these residual surface particles and notches can promote a better mechanical interlocking and interfacial connections between the wind blade fibres and the hydrated concrete mortar, which can have a positive impact on the mechanical properties. In addition, the relatively wider and deeper lanes can provide effective nucleation sites, thus promoting the formation of hydration products near the fibres [64]. However, compared to pure glass fibres, the mechanical treatment process can cause damage, such as roughness on the surface of the WIC fibres, and it is unclear whether the detectable damage limits the strength or to what extent it leads to the weakening of the recovered fibres. Overall, the indications are that the mechanical properties of the WIC improved.

Representative SEM images of the fracture surfaces of WIC-1, WIC-1.5, and WIC-2 samples are shown below. First, the interfacial transition zone (ITZ) boundary between the paste and aggregate of the WIC-1 sample is denser and more uniform. The paste and aggregate are fused, observing each sample’s microstructure. The fibres are well impregnated by the matrix, with few apparent pores. The other samples have obvious micro-cracks, which may be caused by the shrinkage of the hardened paste or the expansion of gaps in the hardened paste, and the matrix around the ITZ shows a laxity, with a large amount of granular material filled in between the lamellar structures, and a lack of continuity between the particles. This may be one of the most important reasons for the high compressive strength of the WIC-1 sample. It can be concluded from the SEM images that the interface between the wind turbine blades of WIC-1 and the mortar matrix is tightly bonded. The fibres are firmly embedded in the matrix. The strongly bonded interface can transfer the stresses to the highly ductile fibres when the it faces external forces [38]. From the distribution of wind blade fibres in WIC-1, on the one hand, most of the wind turbine fibres are single fibres, and the distribution is relatively uniform, forming an interlocking three-dimensional mesh structure, which effectively hinders the stress propagation; on the other hand, the large specific surface area of single fibres increases the contact area with the matrix, which is conducive to the ITZ. In contrast, the ITZ of the WIC-2 sample is weaker. Although the WIC fibre incorporation was high, the distribution was not uniform. At the same time, adding fibres reduces the mortar’s compactness, and increasing the fibre content will result in a higher porosity, leading to a rapid decrease in the compressive strength. Comparatively, the microscopic characterisation of WIC-1.5 is in the middle of the two. This can explain the higher compressive strength of WIC-1 compared to that of WIC-2.

**Figure 14:**
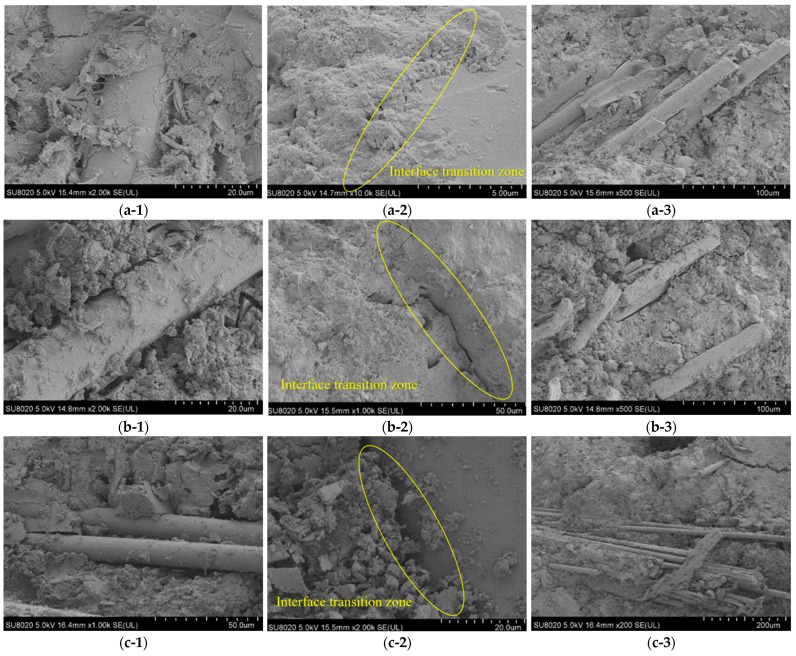
SEM images: (**a-1**–**a-3**) WIC-1; (**b-1**–**b-3**) WIC-1.5; and (**c-1**–**c-3**) WIC-2.

Figure 14(a-3,b-3) show that the WIC-1 and WIC-1.5 samples showed more minor cracks after stress, which were bridged by the long fibres to inhibit the final damage. Some of the fibres were pulled out due to the weak interfacial strength. This may be due to changes in the epoxy residue on the fibre surface, which weakens the molecular interactions and hydrogen bonding that occur at the interface between the pulled fibres and the PLA [64]. Also, the fibres of WIC-1 and WIC-2 in the images are dispersed in short bundles with a higher degree of fragmentation, which may loosen the microstructure of the mortar matrix. This should be one of the reasons for the lower flexural strength of WIC-1. According to the literature, the higher the fibre content, the more pronounced the bridging effect of the fibres [65]. The presence of large and small pores in the structure of WIC-2 is shown in Figure 14(c-3). However, the fibres form a holding framework, and the mesh structure is quite dense, which allows the slurry to penetrate, thus providing a good bond between the fibres and the matrix and stabilising the microstructure of the matrix. This explains the enhanced flexural strength of WIC-2. Another reason is the uneven surface of the WIC fibres, which increases their difficulty slipping from the mortar matrix [60]. Highly doped fibres support the skeleton at various places, filling the “big holes” and “small gaps” and strengthening the continuity and integrity of the matrix. When it faces external forces, the fibres can absorb part of the external energy, which is not easy to pull out, to transfer the load bridge function. The contact area between the highly doped wind blade fibres and the mortar matrix is extensive, and the holding force is more potent, which improves the flexural strength of the composite material [53].

#### 3.3.2. XRD

**Figure 15:**
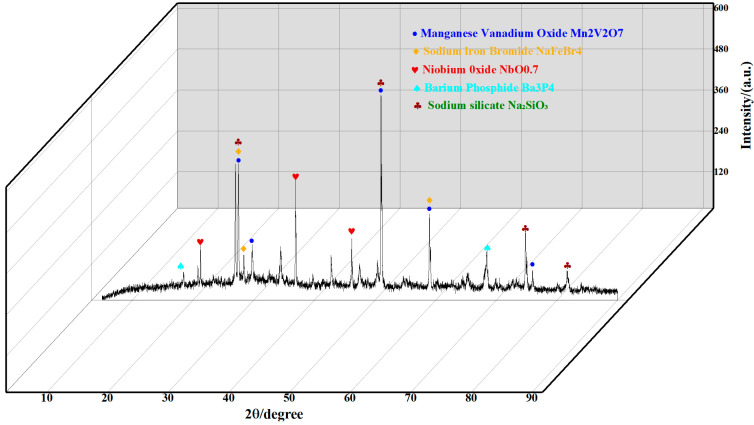
XRD test results.

Figure 15 shows the XRD analysis results of the WIC samples. Using Bragg’s law and the Debye–Scherrer equation, the fibre interlayer distance (d) and crystal parameters (Lc and La) can be calculated to analyse the crystalline properties of the WIC fibres.

(13) d=λ/(2sin⁡θ)

(14) L=Kλ/(βcos⁡θ)

where “l” is the wavelength of CuKa radiation (0.154 nm) and q is the Bragg diffraction angle in radians. b is the full width at half-maximum (FWHM) at reflection, and K is the Scherrer shape factor of the peaks, which was taken to be 0.89 and 1.84, according to literature [21].

According to the calculations, the interlayer spacing of the wind blade fibres is lower than the equivalent value of 0.355 nm for the original fibres, indicating that the layer structure has a better alignment towards the fibre axes [66]. The SEM image above also provides testimony of this.

A sharp peak at 48.5° can be observed with crystalline sodium silicate (NaSiO_3_) and manganese vanadium oxide (Mn_2_V_2_O_7_). The addition of special additives or materials during concrete production causes the presence of manganese vanadium oxide, which plays a catalytic role in WIC. The existence of fibre silicones is due to the sodium silicate-based epoxy resin adhering to the surface of the wind turbine blade fibres. Once again, it is demonstrated that the wind blade fibres are mainly composed of glass fibres with epoxy resin, which are wrapped or bound by the epoxy resin. This is consistent with the results of the SEM images.

## 4. Recycled Concrete Intrinsic Model and Prediction

**Figure 16:**
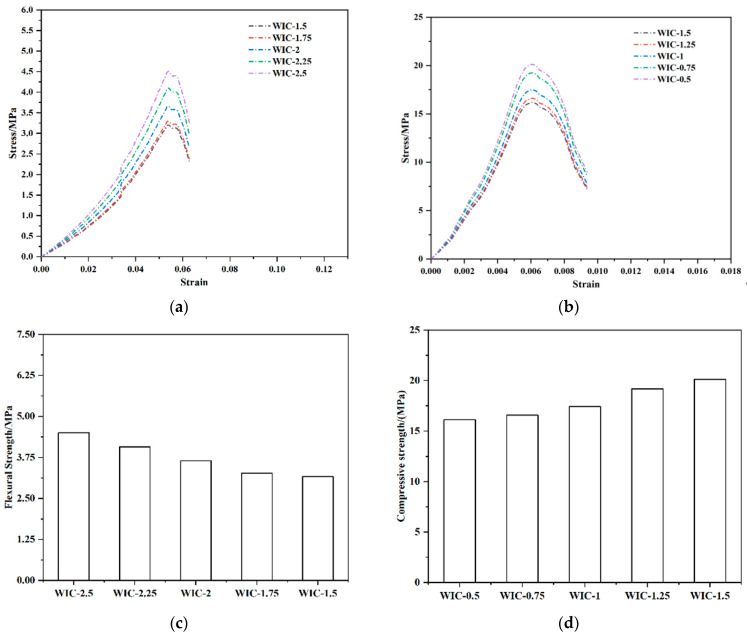
Finite element prediction results for WIC: (**a**) flexural stress–strain curve; (**b**) compressive stress–strain curve; (**c**) histogram of compressive strength; and (**d**) histogram of flexural strength.

Figure 16a,b show the comparison of the measured stress–strain curves with the simulated stress–strain curves for different fibre types and fibre dosages. It can be seen from the figure that the Z-W-T constitutive model can be used to fit the stress response of the fibre concrete material under a higher strain rate better. Meanwhile, not only does the Weibull damage model provide a better fit to the linear elastic cross-section of the stress–strain curve, but also could fit better the plastic and softening segments of the stress–strain curve of the material, which fully describes the stress response of WIC.

The above analyses show that the experimental results of this test are more compatible with the predicted calculation results of the intrinsic model, which examines and verifies test data’s excellent effect, validity, and accuracy. To explore more intensively the change rule of concrete strength with the change in fibre and to summarise the results of this test, we decided to use the finite element model to infer and predict the strength of WIC. The concrete model with 0.5–2.25% wind blade fibre admixture was set up based on the original data at intervals of 0.25%, and the stress–strain curves predicted by the intrinsic relationship are shown in Figure 16. The flexural strength of WIC increases with the enhancement in the fibre amount and the flexural strength decreases with the enhancement in the fibre amount. The results show that WIC has high mechanical properties, which promote elastic deformation while increasing strength.

## 5. Conclusions

In this research, based on the physical recycling method of used wind turbine blades, recycled fibres were obtained through a unique process to explore the effect on the mechanical properties based on the comparison of GC and WIC, as well as the comparison of NAC and RAC. The main conclusions are as follows:

1. The compressive strength of concrete was increased by adding an appropriate content of wind blade fibres. With the addition of wind blade fibres, the decreasing branch of the compressive stress–strain curve tends to flatten; so, the higher the addition of wind blade fibres, the higher the toughness. The specimens containing wind blade fibres have ductility compared to the glass fibre group. The compressive strength of GC was found to be higher than that of WIC at a higher admixture (2%); however, the gain in the compressive strength of WIC was better at a lower admixture (1%).
2. The wind blade fibres can significantly enhance the flexural strength. The stress–strain curves of the control specimens showed a sharp decrease to zero along the linear ascending branches until the concrete cracked. In contrast, the wind blade fibre specimens showed hardened or softened branches along the linear ascending branches. This characteristic of and the enhancement in the concrete properties tends to be more significant when there is a higher content of wind blade fibres. Although the flexural strength of WIC is lower than that of GC, the strength gain for the plain concrete is up to 18.5%.
3. For the peak compressive and flexural strengths, the reinforcing effect of wind blade fibres on concrete is significantly weaker than that of glass fibres. However, the surface of the recycled fibres recovered from wind turbine blades is more likely to have a resin coating, which provides additional slip resistance at the fibre–cement interface and improves the interlocking properties. Thus, the wind blade fibres also have a significant reinforcing effect on plain concrete. The strength of this effect is related to the random distribution of the fibres. This indicates that the effect of WIC fibres is comparable to or better than that of glass fibres in the appropriate dosage range, which proves the feasibility and practicality of the fibres prepared by this recycling method.
4. Although recycled aggregates have been studied extensively, the combination of recycled aggregates with recycled fibres is not common. Even when recycled fibres were added to the second-generation RAC, the strength was significantly lower than that of the NAC group. The RCAs derived from the RAC reduced the strength of the concrete, and the gain effect of the fibres was lower than this reduction effect. This informs the subsequent selection of recycling methods for materials.
5. The SEM results showed that the high-strength WIC presented a denser and more homogeneous ITZ, and it could be observed that the fibres were tightly bonded to the mortar matrix, with a more pronounced bridging effect; in contrast, fine cracks could be observed in the low-strength WIC, and the ITZ presented a relaxed state. In addition, the wind blade fibres are basically monofibres with a uniform distribution and large specific surface area, which increases the contact area with the matrix and facilitates the ITZ, verifying the gain effect. The XRD analysis results show that the WIC fibres are composed of glass fibres and epoxy resin, with a better alignment of the layer structure with the fibre axis.
6. There are still some limitations to this experiment. For example, wind power fibres are organic materials that are usually not alkali-resistant and concrete is alkaline, which may adversely affect the performance. In subsequent studies, methods to address this issue need to be considered, such as selecting suitable reagents, improving the formulation, or chemically modifying the wind power fibres to enhance their stability and durability in alkaline environments.

## Figures and Tables

**Table 1:** Physical properties of P·O 42.5 cement.

Standard Consistency Water Consumption/%	Density/(g/cm^3^)	Heat Loss/%	Solidification Time/min		Stability	Flexural Strength/MPa	
			Initial	Final		3 Day	28 Day
28.2	3.11	3.76	165	305	eligible	5.5	8.6

**Table 2:** Main chemical composition of P·O 42.5 cement (%).

C_3_S	C_2_S	C_4_AF	C_3_A	f-MgO	f-CaO	Else
60.3	19.3	8.6	7.0	2.0	0.7	2.1

**Table 3:** Properties of coarse aggregates.

Crushed Stone Specifications/mm	Bulk Density/(kg/m^3^)	Apparent Density/ (kg/m^3^)	Crushing Value/%	Granule Content of Needle Chips/%	Porosity/%	Mud Content/%
5–26.5	1460	2661	11.2	4.2	43	0.6

**Table 4:** Fine aggregate properties.

Raw Materials	Apparent Density/(kg/m^3^)	Capacity/(kg/m^3^)	Water Absorption/%	Crush Value/%
Natural medium-grained Sand	2689	1655	0.51	/
RCA	2603	1408	6.7	14.9

**Table 5:** Average statistical characteristics of recycled wind blade fibres.

Length/mm	Width/mm	Thickness/mm	Aspect Ratio
25.6	5.2	0.65	5.6

**Table 6:** Physical properties of glass fibres.

Density/(g·cm^3^)	Line Density/dtex	Calibre/μm	Length/mm	Tensile Strength/MPa	Rupture Expansion Rate/%	Tensile Modulus/GPa
2.7	78	15	3, 6, 9, 12	2000	2.5	75

**Table 7:** Concrete mix ratio-1.

Original Concrete	OG
Glass fibre (1%) concrete	GC-1
Glass fibre (2%) concrete	GC-2
Wind blade recycled fibre (1%) concrete	WIC-1
Wind blade recycled fibre (1.5%) concrete	WIC-1.5
Wind blade recycled fibre (2%) concrete	WIC-2
Wind blade fibre (1%) recycled aggregate (20%) concrete	WIC-1RAC20
Wind blade fibre (1.5%) recycled aggregate (20%) concrete	WIC-1.5RAC20
Wind blade fibre (2%) recycled aggregate (20%) concrete	WIC-2RAC20
Wind blade fibre (1%) recycled aggregate (40%) concrete	WIC-1RAC40
Wind blade fibre (1.5%) recycled aggregate (40%) concrete	WIC-1.5RAC40
Wind blade fibre (2%) recycled aggregate (40%) concrete	WIC-2RAC40

**Table 8:** Concrete mix ratio-2.

Groups	Water/(kg/m^3^)	Cement/(kg/m^3^)	Coarse Aggregates (5–10 mm)/(kg/m^3^)	Coarse Aggregates (10–20 mm)/(kg/m^3^)	RCA/(kg/m^3^)	Natural Medium Sand/(kg/m^3^)	Fibre/(g)
OG	195	487	341	797	-	613	0
GC-1	195	487	341	797	-	613	315.33
GC-2	195	487	341	797	-	613	637.27
WIC-1	195	487	341	797	-	613	315.33
WIC-1.5	195	487	341	797	-	613	478.13
WIC-2	195	487	341	797	-	613	637.27
WIC-1RAC20	195	487	341	797	153.5	613	315.33
WIC-1.5RAC20	195	487	341	797	153.5	613	478.13
WIC-2RAC20	195	487	341	797	153.5	613	637.27
WIC-1RAC40	195	487	341	797	408.5	613	315.33
WIC-1.5RAC40	195	487	341	797	408.5	613	478.13
WIC-2RAC40	195	487	341	797	408.5	613	637.27

**Table 9:** Key results of the compression tests.

Type	f_c_ (MPa)	ε_c_ (%)	E_m_ (GPa)
OG	16.56	0.0071	24.15
GC-1	18.19	0.00903	20.34
GC-2	21.21	0.00909	22.7
WIC-1	19.25	0.00865	22.35
WIC-1.5	18.75	0.00743	24.56
WIC-2.0	17.56	0.00589	29.1
WIC-1RAC20	13.47	0.00903	14.7
WIC-1.5RAC20	12.86	0.00810	15.98
WIC-2RAC20	11.26	0.00631	17.51
WIC-1RAC40	12.26	0.00729	17.33
WIC-1.5RAC40	11.154	0.00662	16.72
WIC-2RAC40	8.017	0.00645	12.84

f_c_: compressive strength; ε_c_: strain at compressive strength; E_m_: modulus of elasticity during compression.

**Table 10:** Key results of the four-point bending tests.

Type	f_c_ (MPa)	ε_c_ (%)	Toughness (J)
OG	3.625	0.0689	0.02842
GC-1	5.102	0.0764	0.01641
GC-2	5.58	0.0790	0.07599
WIC-1	3.748	0.0534	0.0249
WIC-1.5	3.958	0.0669	0.03778
WIC-2.0	4.15	0.0634	0.03029
WIC1-RAC20	3.13	0.1003	0.01241
WIC1.5-RAC20	3.38	0.1122	0.02481
WIC2-RAC20	3.618	0.1117	0.09423
WIC1-RAC40	2.739	0.0991	0.01203
WIC1.5-RAC40	3.13	0.1135	0.03388
WIC2-RAC40	3.45	0.1163	0.04305

f_c_: peak stress; ε_c_: peak strain; toughness: area under the load–deflection curve.

## Data Availability

The original contributions presented in the study are included in the article, further inquiries can be directed to the corresponding author.
